# Symptoms of gastroesophageal reflux disease predicts low voltage zones in the posteroinferior left atrium in patients with persistent atrial fibrillation

**DOI:** 10.1016/j.hroo.2024.05.001

**Published:** 2024-05-14

**Authors:** Astrid Paul Nordin, Emmanouil Charitakis, Carina Carnlöf, Finn Åkerström, Nikola Drca

**Affiliations:** ∗ Heart and Lung Disease Unit, Department of Medicine, Huddinge, Karolinska Institutet, Stockholm, Sweden; † Department of Cardiology, Karolinska University Hospital, Stockholm, Sweden; ‡ Department of Cardiology, Linköping University Hospital and Linköping University, Linköping, Sweden

**Keywords:** Atrial fibrillation, Gastroesophageal reflux disease, Catheter ablation, Voltage mapping, Low voltage zone

## Abstract

**Background:**

The presence of low voltage zones (LVZs) in the left atrium (LA) is associated with the recurrence of atrial fibrillation (AF) after pulmonary vein isolation. Numerous studies have posited a link between gastroesophageal reflux disease (GERD) and AF, attributing this relationship to the anatomical proximity of the esophagus to the posteroinferior wall of the LA.

**Objective:**

The objective of this study was to investigate whether GERD can predict the presence of LVZs in the posteroinferior wall of the LA.

**Methods:**

Five hundred fifty-one patients with persistent AF, scheduled for their first AF ablation procedure, were prospectively enrolled. Voltage maps were collected using a multipolar catheter, and *LVZs* were defined as areas measuring ≥3 cm^2^ with a peak-to-peak bipolar voltage of <0.5 mV. Information on GERD symptoms was collected from the participants through a self-administered questionnaire.

**Results:**

Long-standing persistent AF was present in 22.3% of the total cohort. GERD was present in 29% of patients and LVZs in the posteroinferior wall in 12.7%. In the multivariable analysis, patients with GERD were found to have more than twice the odds (odds ratio 2.26; 95% confidence interval 1.24–4.13; *P* = .008) of exhibiting LVZs in the posteroinferior wall of the LA than patients without GERD. GERD was not associated with LVZs in any other region of the LA.

**Conclusion:**

GERD was found to be independently associated with LVZs in the posteroinferior LA. This association may be attributable to inflammation and may partly explain the link between GERD and AF.

## Graphical abstract

**Figure:**
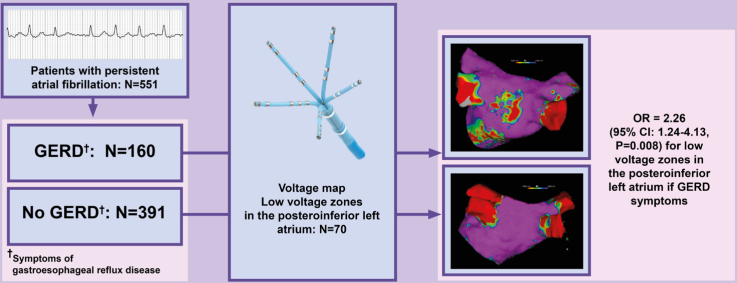

**Key Findings**

▪ A link has been suggested in previous studies between gastroesophageal reflux disease and atrial fibrillation.
▪ Low voltage zones in the left atrium is a well-known predictor of poor outcome regarding arrhythmia freedom after pulmonary vein isolation.
▪ Our findings indicate that gastroesophageal reflux disease is an independent predictor of low voltage zones in the posteroinferior wall of the left atrium in patients with persistent atrial fibrillation, which can partly explain the observed link between gastroesophageal reflux disease and atrial fibrillation.

## Introduction

Atrial fibrillation (AF) is the most prevalent arrhythmia globally.^1^ Numerous conditions predispose individuals to the development of AF, including advanced age, male sex, obesity, hypertension, heart failure, and valvular disease.^2^ Pulmonary vein isolation (PVI) is the cornerstone of catheter ablation of AF and is effective in treating paroxysmal AF, with a success rate of ∼80%.^3^ However, the success rate is lower for patients with persistent AF, at ∼60%.^4^^,^^5^ Atrial fibrosis plays a crucial role in both the initiation and the maintenance of AF.^6^^,^^7^ Previous studies have demonstrated that the presence of low voltage zones (LVZs) in the left atrium (LA) is associated with a lower success rate after PVI,^8^, ^9^, ^10^ and electroanatomic voltage mapping of the LA shows that LVZs can serve as a surrogate indicator for fibrosis.^11^ *Gastroesophageal reflux disease* (GERD) is defined by the presence of troublesome symptoms of heartburn or regurgitation.^12^ The LA posteroinferior wall is in close anatomical proximity to the esophagus,^13^ and multiple studies have shown that GERD appears to predict the development of AF.^14^^,^^15^ However, there is no convincing evidence that patients with GERD exhibit a higher risk of having LVZs in the posteroinferior part of the LA. This study aimed to investigate whether GERD is an independent predictor of the existence of LVZs in the posteroinferior wall of the LA.

## Methods

### Study setting and patients

This observational cross-sectional study included 552 patients scheduled for first-time AF ablation. Patients included in the study were 18 years or older and had persistent or long-standing persistent AF as defined by the European Society of Cardiology guidelines.^2^ Exclusion criteria were an LA diameter of >55 mm (measured in a parasternal long axis in transthoracic echocardiography); acute coronary syndrome during the previous 12 weeks; severe aortic or mitral valve disease; adult congenital heart disease; previous percutaneous or surgical AF ablation, or atrioventricular junction ablation; previous surgery including the left or right atrium; any medical condition likely to limit survival to <1 year; contraindication for oral anticoagulants; AF due to reversible condition; pregnancy; or the participant’s inability or unwillingness to provide informed consent. All participants were consecutively included from May 2020 to October 2023 at the Department of Electrophysiology at both Karolinska University Hospital in Stockholm, Sweden, and Linköping University Hospital in Linköping, Sweden. Information on GERD symptoms was collected from the participants through a self-administered questionnaire about the presence and severity of troublesome symptoms of GERD, such as heartburn or regurgitation.^16^ The study protocol and all data collection were approved by the Swedish Ethical Review Authority (no. 2019-05251), and patient data were collected in accordance with the Declaration of Helsinki. All participants provided written informed consent, and study data were pseudonymized and stored in Research Electronic Data Capture (REDCap), version 11.1.15, a secure electronic data capture tool hosted at Karolinska Institutet.^17^^,^^18^

### Collection of voltage maps

The electrophysiology procedures were performed in line with conventional and local standards. All patients were on oral anticoagulants for a minimum of 3 weeks before the ablation procedure. In addition, they underwent transesophageal echocardiography or computed tomography (CT) (in a minority of the patients) to rule out the presence of thrombus in the left atrial appendage before the procedure. During the procedure, heparin was administered to keep the activated clotting time at >300 seconds. The anatomic maps were collected using a 3-dimensional electroanatomical system (CARTO3, Biosense Webster, Inc., Diamond Bar, CA) with a multipolar catheter after transseptal access. The multipolar catheters used in the study were either 5-splined, each with four 1 mm wide electrodes and 2-6-2 mm spacing (PentaRay, Biosense Webster), or 8-splined, each with four 1 mm wide electrodes and spacing of either 2-2-2, 2-5-2, or 3-3-3 mm (OctaRay, Biosense Webster). Voltage maps were collected during coronary sinus pacing at 600 ms, either before or after PVI; importantly, the operator was blinded to the voltage map before PVI, regardless of whether it was collected before or after the procedure. If AF was present at the beginning of the procedure, electrical cardioversion was performed either before or after PVI. Cardioversion was unsuccessful in 2 patients, who were subsequently excluded. The CONFIDENSE Module (Biosense Webster) was used for continuous mapping, with a manual interpretation of all points to ensure adequate tissue contact. The settings used were 2%–5% cycle length filtering, 4 ms local activation time stability, 4 mm position stability, maximum density, and tissue proximity index activated. If LVZ < 0.5 mV was detected close to the pacing pool of the CS catheter, the bipolar voltage was confirmed during sinus rhythm. If an LVZ was detected, the area was controlled with the ablation catheter (SmartTouch, Biosense Webster or QDot, Biosense Webster) to ensure adequate tissue contact. Only points located outside the pulmonary vein ablation lines were analyzed.

### Analysis of voltage maps

**Figure 1:**
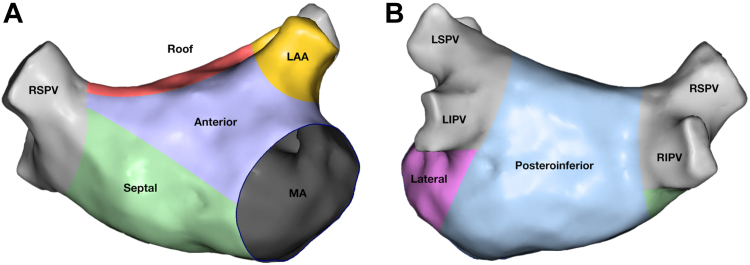
The anatomical segmentation of the left atrium.

Voltage maps were analyzed across all regions of the LA, and the LA was segmented into 6 anatomical zones according to a modified version of the Yagishita model^19^ (Figure 1). The zones were the posteroinferior, lateral, anterior, roof, septal, and left atrial appendage. The analysis of the voltage map was performed after wide antral circumferential ablation, meaning that LVZs located within the wide antral circumferential ablation lines were not measured. An LVZ was deemed significant if it comprised a contiguous area of at least 3 cm^2^, with a peak-to-peak electrogram voltage of <0.5 mV.

### Statistical analysis

Normally distributed continuous variables are presented as mean ± SD. A 2-tailed Student *t* test was used to assess the statistical significance of continuous variables. Categorical variables are presented as count and percentage, and statistical significance was assessed using the Pearson χ^2^ test or Fisher exact test, as appropriate. Variables not normally distributed are presented as median and interquartile range (IQR). The Wilcoxon rank sum test was used to test for statistical significance in these cases. We classified GERD as a dichotomous variable, categorizing it as either “yes” or “no.” Binary logistic regression, which included both univariable analysis and multivariable backward logistic regression analysis, was used to identify whether GERD was an independent predictor of significant LVZs in the posteroinferior regions of the LA. Other significant variables were considered as covariates in the analysis. Covariates with a *P* value of <.1 were included in the multivariable analysis. Variables were assessed for collinearity, and no significant correlation was found. The association of GERD with the presence of LVZs in other regions of the LA was also evaluated. This approach was taken to verify that the presence of LVZs in the posteroinferior region was not merely a result of random chance. A sensitivity analysis was also performed, focusing solely on cases performed exclusively with the PentaRay catheter. A Kruskal-Wallis analysis was performed to compare the degree of GERD symptoms with the size of LVZs, followed by a Mann-Whitney post hoc test. All tests were 2-sided, and a *P* value of <.05 was considered statistically significant. All analyses were performed using STATA/SE version 16.1 (Stata Corporation, College Station, TX).

## Results

### Patient characteristics

We consecutively enrolled 551 patients diagnosed with long-standing persistent (22.3%) or persistent (77.7%) AF, who were scheduled for their first AF ablation procedure. The mean age of the participants was 65.6 ± 8.5 years, the median CHA_2_DS_2_-VASc score was 2 points (IQR 1–3 points), and 26.1% of the cohort were female. In addition, 29% of patients reported GERD symptoms (Table 1). In comparing patients with and without GERD, we found that advanced age, higher CHA_2_DS_2_-VASc score, obstructive sleep apnea syndrome (OSAS), present or previous smoking, and use of proton pump inhibitors (PPIs) were more prevalent in the GERD group.

**Table 1:** Baseline characteristics of patients

Characteristic	All (N = 551)	GERD (n = 160)	No GERD (n = 391)
Age (y)	65.6 ± 8.5	66.9 ± 7.5	65.1 ± 8.8
Sex: female	144 (26.1)	47 (29.4)	97 (24.8)
BMI (kg/m^2^)	27.6 ± 3.7	27.9 ± 3.7	27.5 ± 3.7
CAD	48 (8.7)	14 (8.8)	34 (8.7)
History of congestive heart failure	146 (26.6)	40 (25)	106 (27.1)
Hypertension	323 (58.6)	101 (63.1)	222 (56.8)
Diabetes mellitus	49 (8.9)	20 (12.5)	29 (7.4)
Previous stroke/TIA	52 (9.4)	21 (13.1)	31 (7.9)
CHA_2_DS_2_-VASc score	2 (1–3)	2 (1–3)	2 (1–3)
COPD	22 (4.0)	8 (5.0)	14 (3.6)
OSAS	117 (21.2)	43 (26.9)	74 (18.9)
eGFR (mL/(min·1.73 m^2^))	67.7 ± 12.5	66.9 ± 12.8	68.0 ± 12.4
Long-standing persistent AF	123 (22.3)	36 (22.5)	87 (22.3)
LVEF (%)	51.8 ± 7.6	51.6 ± 7.5	51.5 ± 7.4
LAVI (mL/m^2^)	43.7 ± 11.2	44.5 ± 11.3	43.4 ± 11.1
Present or previous smoking	237 (43.0)	83 (51.9)	154 (39.4)
Present use of alcohol	419 (76.2)	117 (73.1)	302 (77.2)
Use of PPI at baseline	80 (14.5)	49 (30.6)	31 (7.9)

Values are presented as mean ± SD, median (interquartile range), or n (%).

AF = atrial fibrillation; BMI = body mass index; CAD = coronary artery disease; COPD = chronic obstructive pulmonary disease; eGFR = estimated glomerular filtration rate; GERD = gastroesophageal reflux disease; LAVI = left atrial volume index; LVEF = left ventricular ejection fraction; OSAS = obstructive sleep apnea syndrome; PPI = proton pump inhibitor; TIA = transient ischemic attack.

### Voltage maps

All voltage maps were analyzed after PVI, and only the LVZs outside the ablation lines were analyzed. An example of LVZ in the posteroinferior wall is presented in Online Supplemental Figure S1. PentaRay was used in 509 cases (92.4%), with a mean number of mapping points at 1790 ± 1048. OctaRay was used in 42 cases (7.6%), where the mean number of mapping points was 3098 ± 1841. The overall prevalence of LVZs in the LA was 24.5%, with the anterior wall being the most common location for LVZs. The prevalence of LVZs in the posteroinferior wall was 12.7%, and the median area of these was 8 cm^2^ (IQR 5.6–11.5 cm^2^) (Table 2).

**Table 2:** Distribution and size of LVZs

Variable	Prevalence of LVZ ≥ 3 cm^2^ (0.2–0.5 mV)	Size of LVZ (cm^2^)
LA	135 (24.5)	12.2 (7.1–23.4)
AW	101 (18.3)	8.5 (65.9–12.7)
Septum	61 (11.1)	6.3 (4.2–11.3)
PW	54 (9.8)	6.7 (4.6–10.0)
Roof	34 (6.2)	4.2 (3.2–5.4)
IW	35 (6.4)	6.5 (4.3–9.9)
LW	5 (0.9)	4.0 (3.6–4.4)
LAA	1 (0.2)	3.3
PIW	70 (12.7)	8.0 (5.6–11.5)

Values are presented as median (interquartile range) or n (%).

AW = anterior wall; IW = inferior wall; LA = left atrium; LAA = left atrial appendage; LVZ = low voltage zone; LW = lateral wall; PIW = posteroinferior wall; PW = posterior wall.

### GERD as a predictor of LVZs in the posteroinferior wall

GERD was significantly correlated with the existence of LVZs in the posteroinferior wall. After univariable analysis, the presence of GERD was associated with an odds ratio (OR) of 2.32 (95% confidence interval [CI] 1.39–3.77; *P* = .003). After adjustment for age and sex, GERD was still associated with LVZs in the posteroinferior wall with an OR of 2.17 (95% CI 1.27–3.71; *P* = .005). Further adjustment for age, sex, coronary artery disease, hypertension, OSAS, left atrial volume index, estimated glomerular filtration rate, alcohol use, and use of PPIs did not change the OR significantly (OR 2.26; 95% CI 1.24–4.13; *P* = .008) (Table 3). The univariable analysis for all covariates is presented in Online Supplemental Table S1. OSAS and alcohol consumption were identified as negative covariates in the analysis. No association was observed between GERD and LVZs in other parts of the LA. This finding held true both when comparing groups with and without GERD (Online Supplemental Table S2) and in the multivariable analysis. Given the different types of mapping catheters comprised with different electrode size and spacing, which might affect signal amplitude, we performed a sensitive analysis focusing exclusively on maps acquired with the PentaRay catheter. This analysis resulted in a multivariable model in which GERD remained a significant predictor of LVZs in the posterior wall (Online Supplemental Table S3). When comparing the degree of GERD symptoms to sizes of LVZs, we observed a significant difference between patients without GERD symptoms and those with mild or moderate to severe symptoms. However, no significant differences were detected between patients with mild symptoms and those with moderate to severe symptoms.

**Table 3:** GERD as a predictor of LVZs in the posteroinferior wall

Variable	Univariable analysis	Age-adjusted OR	Multivariable OR∗	Multivariable OR†
Patients without LVZs in PIW	1.00	1.00	1.00	1.00
Patients with LVZs in PIW	2.32 (1.39–3.87)	2.22 (1.30–3.77)	2.17 (1.27–3.71)	2.26 (1.24–4.13)
*P*	.001	.003	.005	.008

Values are presented as OR (95% CI) unless specified otherwise.

CAD = coronary artery disease; CI = confidence interval; eGFR = estimated glomerular filtration rate; GERD = gastroesophageal reflux disease; LAVI = left atrial volume index; LVZ = low voltage zone; OR = odds ratio; OSAS = obstructive sleep apnea syndrome; PPI = proton pump inhibitor; PIW = posteroinferior wall.

∗ Adjusted for age and sex.

† Adjusted for age, sex, CAD, hypertension, OSAS, LAVI, eGFR, alcohol use, and use of PPI.

## Discussion

The most significant finding of this study is that patients with GERD had >2 times higher odds of having LVZs in the posteroinferior wall of the LA. Notably, the prevalence of GERD in our study was high, at 29%, in comparison to the previously described prevalence range of 8.8%–25.9% in Europe. The prevalence of GERD appeared to increase until 1999 but has since stabilized.^20^ The high prevalence of GERD observed in our study population with AF further strengthens the association between GERD and AF, as previously reported by Huang et al.^14^ Several studies have established that LVZs are independent predictors of the recurrence of AF after PVI^8^, ^9^, ^10^; however, the underlying causes of LVZ development are still not well understood. Persistent AF, advanced age, enlarged LA, and female sex have been identified as predictors of LVZs in several studies. In addition, diabetes mellitus, impaired renal function, and elevated levels of N-terminal pro b-type natriuretic peptide have been shown to be predictors in some studies.^21^, ^22^, ^23^, ^24^ The precise mechanisms through which these predictors contribute to the development of LVZs are not fully understood; however, the activation of fibroblasts and the production of collagen are believed to play significant roles.^25^ Other mechanisms suggested for the development of LVZs include hormonal factors^26^ and the presence of excessive epicardial fat, which can lead to local inflammation.^27^ Numerous studies have shown the association between AF and GERD,^14^^,^^15^^,^^28^ and the concept of “cardiogastric interaction” was delineated by Linz et al,^29^ who highlighted the potentially involved mechanisms. These are suggested to encompass local and systemic inflammation, chemical or mechanical irritation, and irritation of the vagal nerve.^29^ Inflammation, including both systemic and local responses, is believed to play a role in the development of LVZs,^30^^,^^31^ and there is a close proximity between the posteroinferior wall of the LA and the esophagus. Notably, the LA wall in the superior part of the posterior section is thinner than that in the inferior part. Furthermore, the closest contact point with the esophageal wall is found in this inferior part, and the fat pad layer situated between the esophagus and the LA is thinner in the inferior part than in the superior part.^13^ In GERD, the mucosa of the esophagus can be adversely affected, leading to functional and structural abnormalities. This condition is often a result of the regurgitation of gastric contents, including acid, gastric pepsin, and gastric and duodenal proteases. Such regurgitation can lead to chronic inflammation, characterized by the release of various proinflammatory mediators.^32^ A study performed by Maret-Ouda et al^28^ showed that inflammation in the esophagus, verified through gastroscopy, was independently associated with AF. Our study revealed an independent association between GERD and the presence of LVZs in the posteroinferior regions of the LA. This finding may lend support to the hypothesis proposed by Linz et al, suggesting that chemical and mechanical irritation from the esophagus could contribute to the development of AF. We also investigated whether GERD was a predictor of LVZs in other regions of the LA and found no association, further supporting our hypothesis.

Several studies have demonstrated that the use of PPIs can reduce the burden of AF. The exact mechanisms behind this effect are not completely understood; however, it is hypothesized that the suppression of oxidative stress and inflammation by PPIs may partially explain this finding.^33^ In our study, a higher number of patients with LVZs in the posteroinferior wall of the LA were found to be using PPIs, likely because of a higher prevalence of GERD. While it is improbable that established LVZs would resolve with the use of PPIs, it remains unclear whether PPIs can prevent the development of LVZs in the posteroinferior wall.

### Clinical implications

Patients with GERD were found to have more than twice the odds of exhibiting LVZs in the posteroinferior wall. This association, along with other risk factors for LVZs, should be taken into account when planning for ablation, given that it may necessitate a more complex procedure and the consideration of radiofrequency ablation. Furthermore, GERD is a condition that can be managed through lifestyle modifications, the use of PPIs, or surgical interventions. Successfully treating GERD may reduce the risk of forming LVZs in the posteroinferior wall, potentially diminishing the subsequent risk of developing AF. However, the effectiveness of this approach needs to be rigorously evaluated through a randomized controlled trial.

### Limitations

One limitation of this study is the method of evaluating GERD, which relied on subjective symptom reporting using a self-evaluated scale, as performed in the Nord-Trøndelag Health Study study.^16^ GERD was not objectively confirmed through gastroscopy or pH measurement in the esophagus in our study. It is crucial to acknowledge that GERD diagnosis commonly relies on subjective symptoms such as heartburn or regurgitation.^34^ However, these symptoms could, in theory, originate from an undiagnosed cardiac condition that may lead to LVZs. This possibility introduces a risk of exposure misclassification. Second, the observational and cross-sectional design of our study means that we did not track patients longitudinally, precluding the establishment of a causal link between GERD and AF recurrence after PVI. Further longitudinal studies are required to elucidate this relationship. However, several previous studies have demonstrated that LVZs are predictors of AF recurrence after PVI. Third, the absence of CT imaging in our study precluded the precise assessment of the esophagus’s location, preventing us from correlating its position with specific LVZs. It is important to note that the esophagus is a mobile structure, which means its location can vary.^35^^,^^36^ Because of the shortage of CT scans, we were also unable to investigate other anatomical structures (eg, aorta) that might theoretically contribute to the presence of LVZs. Lastly, as our study exclusively included patients with persistent AF, the findings are not applicable to those with paroxysmal AF.

## Conclusion

GERD symptoms was found to be independently associated with LVZs in the posteroinferior wall of the LA. This association could be attributed to inflammation, which may offer an explanation for the observed link between GERD and AF. The effectiveness of PPIs in preventing the development of LVZs in the posteroinferior part of the LA remains uncertain.
