# Supplementary material for: Symptoms of gastroesophageal reflux disease predicts low voltage zones in the posteroinferior left atrium in patients with persistent atrial fibrillation
Source: Heart Rhythm O2. 2024 May 14;5(6):351–6. doi: 10.1016/j.hroo.2024.05.001 (PMC11228275; doi:10.1016/j.hroo.2024.05.001)
Supplement: Supplemental Material [file mmc1.docx]

Table S1. Univariable analysis for factors predicting low voltage zones in the left atrial posteroinferior wall.

| Variable | OR [95% CI] | P-value |
| --- | --- | --- |
| Sex (female) | 3.19 [1.90-5.33] | **<0.001** |
| Age (per one year increase) | 1.12 [1.07-1.17] | **<0.001** |
| BMI (kg/m^2^) | 0.97 [0.91-1.04] | 0.44 |
| CAD | 2.56 [1.26-5.10] | **0.009** |
| History of congestive heart failure | 1.03 [0.59-1.82] | 0.91 |
| Hypertension | 1.76 [1.03-3.03] | **0.04** |
| Diabetes mellitus | 1.38 [0.62-3.09] | 0.43 |
| Previous ischemic stroke or TIA | 0.89 [0.36-2.16] | 0.79 |
| CHA_2_DS_2_-VASc (per one point increase) | 1.50 [1.28-1.76] | **<0.001** |
| COPD | 1.56 [0.51-4.75] | 0.46 |
| OSAS | 0.44 [0.20-0.95] | **0.036** |
| GERD | 2.32 [1.39-3.87] | **0.001** |
| Previous or current smoking | 1.47 [0.89-2.44] | 0.13 |
| Use of alcohol | 0.55 [0.32-0.94] | **0.029** |
| Use of PPI at baseline | 2.09 [1.13-3.86] | **0.018** |
| Longstanding persistent AF | 1.10 [0.69-1.97] | 0.76 |
| - LVEF (%) | 1.00 [0.96-1.03] | 0.85 |
| - LAVI (per one ml/m^2^ increase) | 1.04 [1.02-1.06] | **0.001** |
| eGFR (ml/min/1.73 m^2^) per one unit decrease | 1.04 [1.02-1.06] | **<0.001** |

AF: atrial fibrillation, BMI: body mass index, COPD: chronic obstructive pulmonary disease, CAD: coronary artery disease, eGFR: estimated glomerular filtration rate, GERD: gastroesophageal reflux disease, LAVI: left atrial volume index, LVEF: left ventricular ejection fraction, OSAS: obstructive sleep apnea syndrome, PPI: proton pump inhibitor, TIA: transient ischemic attack

Table S2. LVZs in different regions of the LA in patients with and without GERD

|  | GERD | No GERD | P-value |
| --- | --- | --- | --- |
| LA | 40.4% | 22.8% | 0.14 |
| Posteroinferior | 20.0% | 9.7% | **0.001** |
| Posterior | 15.0% | 7.7% | **0.009** |
| Inferior | 11.9% | 4.1% | **0.001** |
| Anterior | 21.2% | 17.1% | 0.26 |
| Septum | 13.1% | 10.2% | 0.33 |
| Roof | 5.6% | 6.4% | 0.73 |
| Lateral | 0.6% | 0.01% | 0.67 |
| LAA | 0.0% | 0.3% | 0.52 |

GERD: gastroesophageal reflux disease, LA: left atrium, LAA: left atrial appendage, LVZ: low voltage zone

Table S3. Sensitivity analysis of GERD as a predictor of LVZs in the posteroinferior wall (OR [95% CI].

Multivariable analysis conducted exclusively on cases utilizing PentaRay.

|  | Univariable analysis | Age-adjusted OR | Multivariable OR† | Multivariable OR‡ |
| --- | --- | --- | --- | --- |
| Patients without LVZ in PIW | 1.00 | 1.00 | 1.00 | 1.00 |
| Patients with LVZ in PIW | 2.33 [1.38-3.95] | 2.18 [1.26-3.74] | 2.13 [1.23-3.69] | 2.21 [1.21-4.05] |
| P-value | 0.002 | 0.005 | 0.007 | 0.010 |

†adjusted for age and sex

‡adjusted for age, sex, CAD, hypertension, OSAS, LAVI, eGFR, alcohol use and use of PPI

CAD coronary artery disease, CI: confidence interval, eGFR: estimated glomerular filtration rate, GERD: gastroesophageal reflux disease, LAVI: left atrial volume index, LVZ: low voltage zone, OR: odds ratio, OSAS: obstructive sleep apnea syndrome, PPI: proton pump inhibitor, PIW: posteroinferior wall

Figure S1. Voltage map showing posteroinferior LVZ.


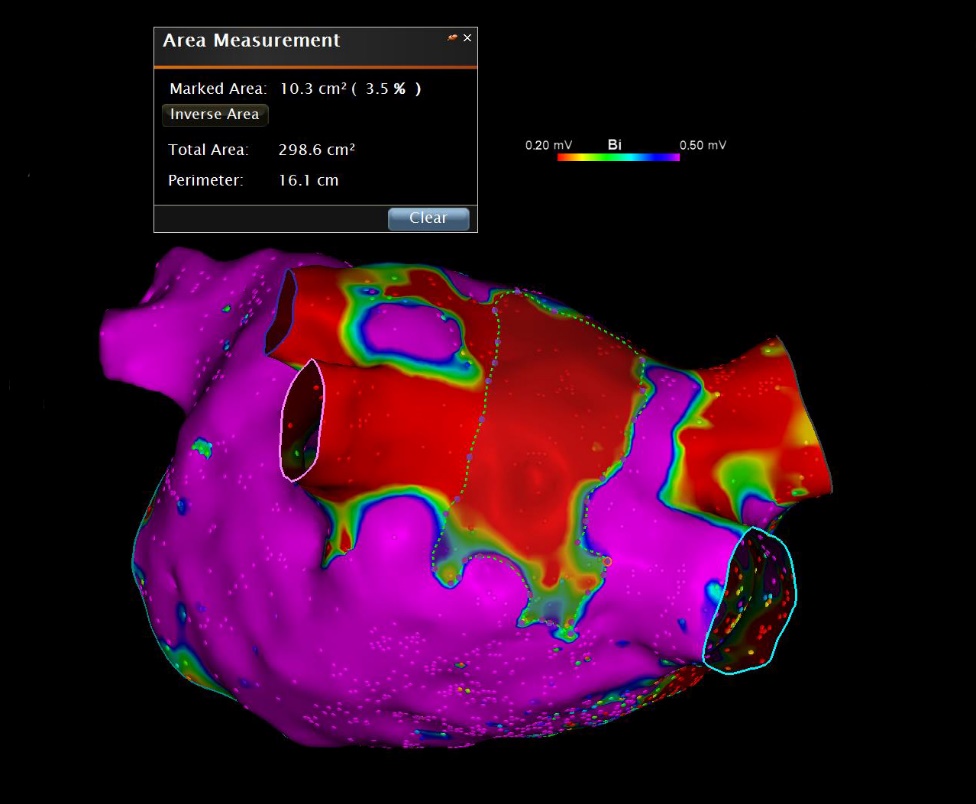


LVZ: low voltage zone
